# Identification of candidate ATP-binding cassette transporter gene family members in *Diaphorina citri* (Hemiptera: Psyllidae) via adult tissues transcriptome analysis

**DOI:** 10.1038/s41598-019-52402-3

**Published:** 2019-11-01

**Authors:** Zhengbing Wang, Fajun Tian, Lijun Cai, Jie Zhang, Jiali Liu, Xinnian Zeng

**Affiliations:** 1 Guangdong Engineering Research Center for Insect Behavior Regulation, Key Laboratory of Bio-Pesticide Innovation and Application, College of Agriculture, South China Agricultural University, Guangzhou, 510642 China; 2 State Key Laboratory of Ecological Pest Control for Fujian and Taiwan Crops, Institute of Applied Ecology, Fujian Agriculture and Forestry University, Fuzhou, 350002 China

**Keywords:** Biochemistry, Transcriptomics

## Abstract

The ATP-binding cassette (ABC) transporters exist in all living organisms and play major roles in various biological functions by transporting a wide variety of substrates across membranes. The functions of ABC transporters in drug resistance have been extensively studied in vertebrates; however, they are rarely characterized in agricultural pests. The Asian citrus psyllid, *Diaphorina citri*, is one of the most damaging pests of the Citrus genus because of its transmission of Huanglongbing, also known as Yellow Dragon disease. In this study, the next-generation sequencing technique was applied to research the ABC transporters of *D*. *citri*. Fifty-three ABC transporter genes were found in the RNA-Seq data, and among these ABC transporters, 4, 4, 5, 2, 1, 4, 18 and 15 ABC proteins belonged to the ABCA-ABCH subfamilies, respectively. Different expression profiles of 52 genes between imidacloprid-resistant and imidacloprid-susceptible strains were studied by qRT-PCR; 5 ABCGs and 4 ABCHs were significantly upregulated in the imidacloprid-resistant strain. In addition, five of the nine upregulated genes were widely expressed in adult tissues in spatial expression analysis. The results suggest that these genes may play key roles in this phenotype. In general, this study contributed to our current understanding of *D*. *citri* resistance to insecticides.

## Introduction

The ATP-binding cassette (ABC) transporter family is one of the largest families of membrane proteins and universally exists in all living organisms on Earth^1^. The first one was found in prokaryotes. In humans, 48 ABC family members have been identified^2^. The majority of these proteins are membrane-bound primary active transporters that transport various molecules across all cell membranes by binding ATP^3^. Based on the components of their ATP-binding domain(s), also known as nucleotide-binding domains (NBDs), they are classified as ABC transporters. Each NBD contains three characteristic motifs: ABC signature C, Walker A box and Walker B box. The function of NBDs is to bind and hydrolyse ATP to provide energy for substrate transportation. In addition, the ABC transporters also contain a transmembrane domain (TMD) which usually consists of five to seven membrane helices and participates in the recognition of the substrates. Some eukaryotic ABC transporters are composed of 2 NBDs and 2 TMDs, known as full-transporters, whereas those with only 1 NBD and 1 TMD are called half-transporters. The latter require either homodimers or heterodimers to form a functional unit^4,5^. According to the homology of the NBD sequences, the ABC transporter family is classified into 8 subfamilies, named ABCA-ABCH.

In recent years, ABC transporters have received increasing attention regarding detoxification. In humans, the overexpression of various ABC transporters in cancer cells can efficiently remove the anticancer drugs from the cells, thus reducing the efficacy of drugs. The development of multidrug resistance (MDR) in cancer cells is one of the major reasons for the failure of cancer chemotherapy^6,7^. In arthropods, ABC transporters are usually associated with insecticide resistance by reducing toxic concentrations in cells/tissues^8–10^. For instance, ABCG4 correlates with Cry1Ac resistance in *Plutella xylostella*^11^. Some genes of the ABCB, ABCC, ABCD and ABCG subfamilies are upregulated in strains of *Laodelphax striatellus* resistant to chlorpyrifos, deltamethrin and imidacloprid^12^. ABC transporters (Mdr50, Mdr65, and Mrp1) are involved in DDT resistance in *Drosophila melanogaster*^13^. In a study of the interaction between permethrin and verapamil (an inhibitor of ABC transporters) in *Anopheles stephensi*, the toxicity of permethrin is increased approximately 5-fold with the inhibition of ABC transporters; concurrently, the expression levels of ABCB6 and ABCG4 are significantly upregulated^14^. It has been reported that ABC transporters are associated with the transport and/or resistance to 27 different insecticides belonging to 9 distinct chemical classes of insecticides (carbamates, macrocyclic lactones, neonicotinoids, organophosphates, pyrethroids, cyclodienes, benzoylureas, phenylpyrazoles, and DDT)^15,16^.

The Asian citrus psyllid (ACP), *Diaphorina citri* (Hemiptera: Psyllidae) feeds on citrus flush and transmits *Candidatus* Liberibacter asiaticus (CLas), a phloem-limited bacterium that infects citrus, that causes Huanglongbing, also known as citrus greening disease or Yellow Dragon disease, a destructive disease of citrus. At present, the application of pesticides is the main way to control the ACP^17,18^. However, the long-term use of chemical insecticide has led to the development of varying levels of resistance to almost all common insecticides in the populations of *D*. *citri* in many citrus producing areas^19–21^. The decreased insecticide sensitivity of *D*. *citri* was related to the increased activities of esterases (ESTs), glutathione S-transferases (GSTs), and cytochrome P450 monooxygenases (P450s)^22–24^. A recent study showed that four P450s, one GST, and one EST of *D*. *citri* were associated with imidacloprid resistance^25^.

The development of insecticide resistance seriously impacts the effectiveness of chemical control strategies. Therefore, it is necessary to comprehensively understand the mechanism of insecticide resistance. However, as xenobiotic transporters, the role of the ABC transporters in insecticide resistance in *D*. *citri* has not been determined. To identify the ABC transporter genes in *D*. *citri*, transcriptome analysis was applied in this study. In total, fifty-three ABC transporter genes were found, which were classified into eight subfamilies (A-H) by phylogenetic analysis. The detailed sequence comparisons of the eight subfamilies with other species (*D*. *melanogaster*, *Bombyx mori*, *Tribolium castaneum*, *Tetranychus urticae*, and *Bemisia tabaci*) shed light on our understanding of the evolution of the ABC transporter family among the six species. In addition, the expression profiles of these genes in imidacloprid-susceptible and imidacloprid-resistant strains were analysed by qRT-PCR. Our results provide valuable information on the mechanism of insecticide resistance in *D*. *citri*, and will facilitate the elucidation of the functions of these genes in this citrus pest.

## Results and Discussion

### Identification of ABC transporters in *D*. *citri*

**Figure 1:**
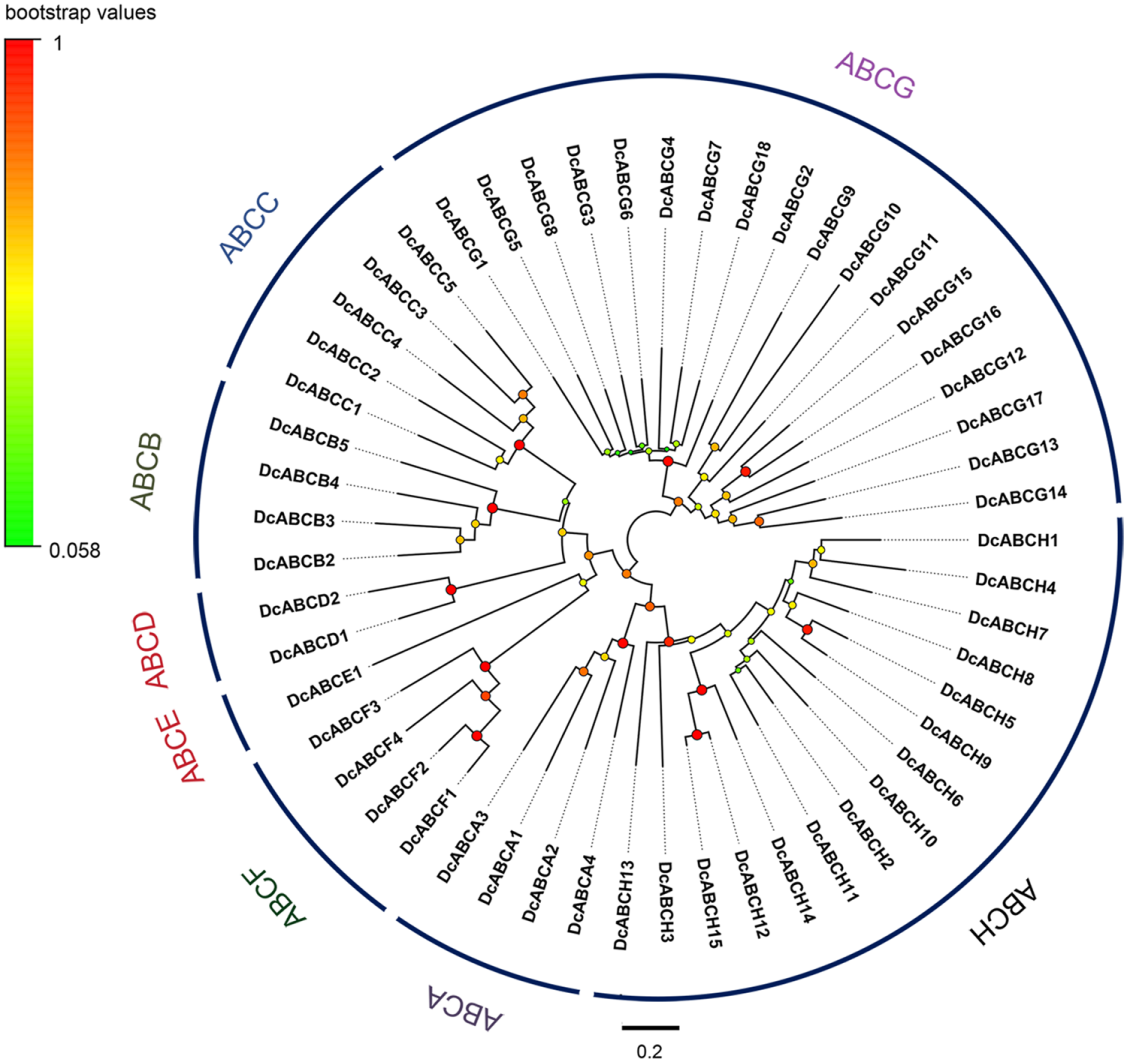
Phylogenetic tree of *D*. *citri* ABC transporters. The amino acid sequences of the nucleotide binding domain (NBD) were used to construct the neighbour-joining tree with the Poisson model. Analysis was performed with MEGA6.0. The bootstrap values resulted from 1000 replications and are displayed in the size and colour of the circles.

**Figure 2:**
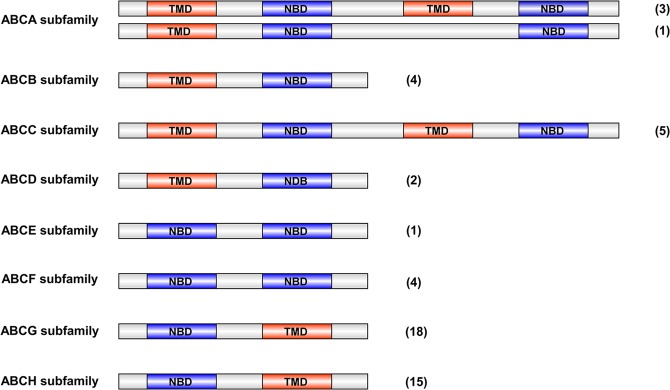
Conserved domain analysis of the ABC transporters of *D*. *citri*. The orange stripes indicate the transmembrane domains, TMDs; the blue stripes represent the nucleotide binding domains, NBDs; the numbers in parentheses indicate the number of ABC transporters in each subfamily.

A total of 53 ABC transporter genes were found in the transcripts of *D*. *citri*, including 52 unigenes with full-length open reading frame (ORF) sequences and the lengths of these ABC transporters ranged from 594 to 2413 amino acids (Table 1). In addition, 91 ABC transporter genes or fragments were also found in the genome of *D*. *citri* (Accession, GCA_000475195.1), and all these genes or fragments can be matched with those identified from the transcriptome data (Table S2). Therefore, we speculate that the 53 ABC transporters we identified are very close to representing all of the ABC transporters. Then, we aligned the NBDs by the ClustalW program and constructed a neighbor-joining tree. According to the homology of the NBDs, these 53 ABC transporters were grouped into the 8 A-H families (Fig. 1). We identified 4, 4, 5, 2, 1, 4, 18 and 15 ABC proteins belonging to the ABCA-H subfamilies, respectively. All genes of subfamilies ABCA and ABCC were full-transporters; in subfamily ABCB, full-transporters were not identified. The ABCD, ABCG, and ABCH subfamilies comprised only half-transporters. However, subfamilies ABCE and ABCF contained only NBDs (Fig. 2). All of the ABC transporter genes of *D*. *citri* were submitted to GenBank (Table 1).

**Table 1:** Characterisation of 53 ABC transporters in *D*. *citri*. aa, amino acid; N-Glc, N-glycosylation sites; O-Glc, O-glycosylation sites.

Subfamily	Name	Accession no.	Length (aa)	Completeness	Matched protein (Accession no.)	Species	E value	N-Glc	O-Glc
A (4)	DcABCA1	MH172490	2413	Complete	AIN44098.1	*Laodelphax striatella*	0	11	8
	DcABCA2	MH172491	1693	Complete	XP_016657979.1	*Acyrthosiphon pisum*	0	6	0
	DcABCA3	MH172492	1674	Complete	XP_022180700.1	*Myzus persicae*	0	0	0
	DcABCA4	MH172493	2001	Complete	XP_023723146.1	*Cryptotermes secundus*	0	8	0
B (4)	DcABCB2	MH172495	830	Complete	XP_021938907.1	*Zootermopsis nevadensis*	0	0	4
	DcABCB3	MH172496	706	Complete	XP_015606144.1	*Cephus cinctus*	0	3	0
	DcABCB4	MH172497	688	Complete	XP_022199620.1	*Nilaparvata lugens*	0	1	0
	DcABCB5	MH172498	644	Complete	XP_018904441.1	*Bemisia tabaci*	0	1	0
C (5)	DcABCC1	MH172499	1523	Complete	XP_025201032.1	*Melanaphis sacchari*	0	5	0
	DcABCC2	MH172500	1407	Complete	XP_021928372.1	*Zootermopsis nevadensis*	0	6	0
	DcABCC3	MH172501	1373	Complete	XP_015366071.1	*Diuraphis noxia*	0	0	0
	DcABCC4	MH172502	1512	Complete	XP_018902692.1	*Bemisia tabaci*	0	4	1
	DcABCC5	MK090470	1343	Complete	XP_018897575.1	*Bemisia tabaci*	0	2	0
D (2)	DcABCD1	MH172503	712	Complete	XP_022192008.1	*Nilaparvata lugens*	0	0	0
	DcABCD2	MH172504	667	Complete	XP_018911183.1	*Bemisia tabaci*	0	2	0
E (1)	DcABCE1	MH172505	610	Complete	XP_023724484.1	*Cryptotermes secundus*	0	0	0
F (4)	DcABCF1	MH172506	608	Complete	KZS19906.1	*Daphnia magna*	0	2	0
	DcABCF2	MH172507	1113	Complete	XP_018327498.1	*Agrilus planipennis*	0	2	1
	DcABCF3	MH172508	629	Complete	XP_025837481.1	*Agrilus planipennis*	0	3	0
	DcABCF4	MH172509	711	Complete	XP_018911520.1	*Bemisia tabaci*	0	2	0
G (18)	DcABCG1	MH172510	612	Complete	XP_018915601.1	*Bemisia tabaci*	0	2	0
	DcABCG2	MH172511	645	Complete	XP_018915017.1	*Bemisia tabaci*	0	0	0
	DcABCG3	MH172512	594	Complete	XP_022181257.1	*Myzus persicae*	0	1	0
	DcABCG4	MH172513	632	Complete	XP_018910658.1	*Bemisia tabaci*	0	1	0
	DcABCG5	MH172514	695	Complete	XP_018907924.1	*Bemisia tabaci*	0	1	2
	DcABCG6	MH172515	711	Complete	XP_018897078.1	*Bemisia tabaci*	0	1	0
	DcABCG7	MH172516	623	Complete	XP_018898633.1	*Bemisia tabaci*	0	0	0
	DcABCG8	MH172517	644	Complete	XP_018897241.1	*Bemisia tabaci*	0	0	2
	DcABCG9	MH172518	627	Complete	XP_018914482.1	*Bemisia tabaci*	0	2	0
	DcABCG10	MH172519	910	Complete	XP_018915492.1	*Bemisia tabaci*	0	1	0
	DcABCG11	MH172520	728	Complete	XP_024214083.1	*Halyomorpha halys*	0	0	0
	DcABCG12	MH172521	609	Complete	XP_018911164.1	*Bemisia tabaci*	0	2	0
	DcABCG13	MH172522	649	Complete	XP_018896422.1	*Bemisia tabaci*	0	1	0
	DcABCG14	MH172523	638	Complete	XP_018896422.1	*Bemisia tabaci*	7e-149	3	0
	DcABCG15	MH172524	609	Complete	XP_014276473.1	*Halyomorpha halys*	0	2	0
	DcABCG16	MH172525	646	Complete	XP_014279356.1	*Halyomorpha halys*	0	2	0
	DcABCG17	MH172526	706	Complete	XP_018908689.1	*Bemisia tabaci*	0	3	0
	DcABCG18	MK090471	606	Complete	XP_025412973.1	*Sipha flava*	0	2	0
ABCH (15)	DcABCH1	MH172527	686	Complete	XP_018896133.1	*Bemisia tabaci*	2e-164	2	0
	DcABCH2	MH172528	703	Complete	XP_001945365.2	*Acyrthosiphon pisum*	0	2	0
	DcABCH3	MH172529	949	Complete	XP_021926127.1	*Zootermopsis nevadensis*	1e-78	7	0
	DcABCH4	MH172530	700	Complete	AKJ85501.1	*Rhopalosiphum padi*	6e-146	1	0
	DcABCH5	MH172531	769	Complete	XP_012522429.1	*Monomorium pharaonis*	0	4	0
	DcABCH6	MH172532	682	Complete	XP_018916054.1	*Bemisia tabaci*	0	1	0
	DcABCH7	MH172533	681	Complete	AKJ85501.1	*Rhopalosiphum padi*	2e-175	3	0
	DcABCH8	MH172534	689	Complete	XP_018896133.1	*Bemisia tabaci*	0	2	0
	DcABCH9	MH172535	764	Complete	XP_025206654.1	*Melanaphis sacchari*	0	2	0
	DcABCH10	MH172536	691	Complete	XP_025208609.1	*Melanaphis sacchari*	0	1	0
	DcABCH11	MH172537	685	Complete	XP_022164150.1	*Myzus persicae*	0	4	0
	DcABCH12	MH172538	677	Complete	XP_018917681.1	*Bemisia tabaci*	7e-114	2	0
	DcABCH13	MH172539	717	Complete	XP_012522429.1	*Monomorium pharaonis*	6e-165	1	0
	DcABCH14	MH172540	645	N-missing	XP_014274344.1	*Halyomorpha halys*	5e-91	1	0
	DcABCH15	MK090472	676	Complete	XP_025197526.1	*Melanaphis sacchari*	1e-109	2	0

### ABCA subfamily

Four ABCA transporters were identified in *D*. *citri*. Three of them were full-transporters, and one was a single TMD-containing ABCA protein (DcABCA2) (Fig. 2). This subfamily includes the largest ABC transporter, which is encoded by DcABCA1 (2413 amino acids), and in fact, subfamily ABCA transporters are typically the largest among known ABCs^26^. A phylogenetic tree was constructed to support the member position for the DcABCAs (Supplementary Fig. S1). DcABCA1 clustered with tetur25g01640 and human HsABCA1, 2, 4 and 7. DcABCA2 reveals a high bootstrap support with a group that is formed by six ABCAs of *B*. *tabaci*, and then aligned with two ABCAs of *T*. *castaneum*, TcABCA-UA and TcABCA-7A, DmCG31731, BmABC004187 and a sister-group that is formed by seven *T*. *urticae* ABCAs; these ABCAs form a clade. DcABCA3 is placed in the human ABCA3 clade, which comprises a sister-group of TcABCAs. The clade also contains two BmABCAs (BmABC007217 and BmABC007221). DcABCA4 clustered with three insect ABCAs and then aligned with five human HsABCAs (HsABCA5, HsABCA6, and HsABCA8-10).

In humans, ABCA transporters play important functions in lipid transport and metabolism^2^. This includes ABCA1, which transports intracellular cholesterol and phosphatide to lipid-poor apolipoprotein A-I (ApoA-I) to form high-density lipoprotein (HDL)^27^. The expression of ABCA1 in the hippocampus is positively associated with the severity of Alzheimer’s disease (AD)^28^. However, the role of the arthropod orthologues of these human ABCAs is currently unclear, but they might be related to lipid transport based on the high conservation of the structure. Injection of dsRNAs of TcABCA-9A and TcABCA-9B, results in approximately 30% mortality with severe defects in pupae and pharate adults of *T*. *castaneum*^29^.

### ABCB subfamily

The ABCB subfamily contains both full-transporters and half-transporters. In *D*. *citri*, four ABCB transporters were identified, and all of them are half-transporters that comprise one TMD and one NBD. In the phylogenetic tree (Supplementary Fig. S2), the four DcABCBs were allocated to 4 clades. DcABCB2 was clearly clustered with five ABCBs from other species and formed a clade. This clade contains BmABC005473, TcABCB-6A, DmCG4225, Btaq001304.1, and HsABCB6. DcABCB3, DcABCB4, and DcABCB5 are located in three other clades similar to DcABCB2. The half transporters in phylogenetic analysis showed obvious orthologous relationships, suggesting that half-transporters have evolutionarily conserved roles in arthropods^30^.

In humans, HsABCB6-8 and HsABCB10 are four mitochondrial ATP-binding cassette transporters. HsABCB6 is associated with multiple cellular functions, including iron homeostasis and porphyrin transport, and is resistant to several cytotoxic agents^31^. HsABCB7 is associated with Refractory anaemia with ring sideroblasts (RARS)^32^. HsABCB8 is involved in protecting the mitochondrial genome through doxorubicin resistance^33^. HsABCB10 is an important player in the protection from oxidative stress^34^. *D*. *melanogaster* DmCG4225, a homologous gene of *Homo sapiens* HsABCB6 was associated with tolerance to cadmium^35^. DmCG3879 (MDR49) is involved in directing germ cell migration through controlling export of a *Drosophila* germ cell attractant in a signal peptide-independent manner^36^. The homologous gene of HsABCB7 in *Aedes aegypti* is involved in insecticide resistance^37^.

### ABCC subfamily

The C subfamily ABC transporters in humans consist of cystic fibrosis transmembrane conductance regulator (CFTR), membrane-bound sulfonylurea receptors (SURs) and multidrug resistance-associated proteins (MRPs). CFTR (HsABCC7) acts as a chloride channel that is involved in regulating exocrine secretions. SURs (HsABCC8, HsABCC9) binds sulfonylurea and functions as regulators of potassium channels that play a role in modulating insulin secretion. MRPs (HsABCC1-6 and HsABCC10-12) are considered to be important transporters of xenobiotics due to their ability to transport a wide range of substrates (such as drugs, ions, toxins, and endogenous compounds)^2,38,39^. Due to their functions, MRPs are the most well characterized in the ABC transporters subfamily C. All of the human ABCC transporter genes encode full ABC transporters; however, both full- and half-transporters were found in insects^2,40^. A human MRP can be classified as a “long” MRP or “short” MRP based on whether it contains a third N-terminal transmembrane domain (TMD0). If it contains a TMD0, it is considered to be a “long” MRP, (HsABCC1-3, HsABCC6, and HsABCC10); on the contrary, the “short” MRPs include HsABCC4, HsABCC5, HsABCC11, and HsABCC12^41^. In insects, it has been reported that ABCC is involved in insecticide resistance; for instance, when the nymphs of *Nilaparvata lugens* are exposed to triazophos, the transcript level of an ABCC shows a significant increase^42^. In *Pediculus humanus*, silencing PhABCC4 by RNAi leads to an increased susceptibility to ivermectin^43^.

In *D*. *citri*, five ABCC transporter genes were identified; all genes contained full-length ORFs and encoded full ABC transporters (Table 1, Fig. 2). In phylogenetic analysis (Supplementary Fig. S3), DcABCC1 clustered with Btabq019529.2 and Btabq000311.1, DmCG6214, two *B*. *mori* ABCCs, four human MRPs (HsABCC1, HsABCC2, HsABCC3, and HsABCC6), twenty-three *T*. *urticae* ABCCs, and *T*. *castaneum* TcABCC-9A. As an orthologue to human MRPs, DmCG6214 is an ATP-dependent, vanadate-sensitive organic anion transporter and transports developmentally significant hormones, such as ecdysteroid and juvenile hormone^41^. DcABCC2 cluster with three human MRPs (HsABCC5, HsABCC11 and HsABCC12), where HsABCC5 and HsABCC11 act as nucleoside transporters; however, the function of HsABCC12 is unknown^2,44^. DcABCC3 and DcABCC5 were placed in a large clade containing HsABCC4, a large cluster of *T*. *urticae* ABCCs, seven *B*. *mori* ABCCs, a cluster of *T*. *castaneum* ABCC5s, and ten *D*. *melanogaster* ABCCs. HsABCC4 has the ability to transport a wide variety of endogenous and xenobiotic organic anionic compounds out of the cell; these substrates also include molecules involved in cellular signaling^2^. DcABCC4 clustered with human HsABCC10, DmCG7806, BmABC010636, Tetur03g07840, Btabq004618.1, and TcABCC-4A, and this clade showed clear orthologous relationships. HsABCC10 is known as a drug-efflux pump because it is involved in the transport of amphipathic anions, leading to resistance to a variety of anticancer drugs^45^. In the transcriptomes of *D*. *citri*, the orthologues of CFTR and SUR are not identified.

### ABCD subfamily

The ABCD subfamily transporters are half-transporters in animals with one TMD and one NBD and play a role in transporting fatty acids and acyl-CoA in peroxisomes^46^. Two ABCD transporter transcripts were identified in the transcriptomes of *D*. *citri*, both of which have full-length ORFs. The same number of ABCDs was also found in the genome of most other insects^47^ (Table 2). In the phylogenetic tree (Supplementary Fig. S4), *D*. *citri* DcABCD1 clustered with *B*. *tabaci* Btabq026746.1, *T*. *castaneum* TcABCD-6A, *D*. *melanogaster* DmCG2316, *B*. *mori* BmABC004616, *T*. *urticae* tetur05g06640, *H*. *sapiens* HsABCD1 and HsABCD2. DcABCD2 is located in the HsABCD3 clade. The phylogenetic analysis reflected clear orthologous relationships with ABCD transporter proteins among these species, suggesting that ABCD transporters are highly conserved in animals.

**Table 2:** Number of each ABC transporter subfamily in different species.

Species	ABCA	ABCB	ABCC	ABCD	ABCE	ABCF	ABCG	ABCH	TOTAL
*Homo sapiens*	12	11	12	4	1	3	5	0	48
*Daphnia pulex*	3	6	24	3	1	3	26	11	82
*Tetranychus urticae*	9	4	39	2	1	3	23	22	103
*Tigriopus japonicus*	5	5	17	3	1	3	7	5	46
*Brachionus koreanus*	11	19	15	3	1	3	8	2	61
*Drosophila melanogaster*	10	8	14	2	1	3	15	3	56
*Anopheles gambiae*	9	5	13	2	1	3	16	3	52
*Bombyx mori*	6	8	15	2	1	3	13	3	51
*Helicoverpa armigera*	7	11	11	2	1	3	16	3	54
*Plutella xylostella*	15	14	21	3	1	3	19	6	82
*Tribolium castaneum*	10	6	35	2	1	3	13	3	73
*Apis mellifera*	3	7	9	2	1	3	15	3	43
*Bemisia tabaci*	8	3	6	2	1	3	23	9	55
*Lygus hesperus*	11	6	12	2	1	3	19	11	65
*Diaphorina citri*	4	4	5	2	1	4	18	15	53

### ABCE and ABCF subfamilies

The ABCE and ABCF subfamilies are quite distinct from other ABC transporters because they only contain two linked NBDs and lack TMDs (Fig. 2). In view of the special structure, ABCE and ABCF proteins are involved in biological processes other than transportation. RNAi against *Caenorhabditis elegans* ABCE, which is also known as an RNase L inhibitor (RLI) in eukaryotes, resulted in embryonic lethality and slow growth, suggesting that ABCE plays a role in the regulation of translation and transcription^48^. In humans, HsABCE1 has an important role in HIV-1 assembly^49^, and HsABCF1 (ABC50) is associated with promoting translation initiation^50^. In insects, injection of dsRNA specific for *T*. *castaneum* TcABCE-3A and TcABCF-2A, led to 100% mortality in the larvae of *T*. *castaneum*^29^.

One ABCE and four ABCF transporter genes were identified in the *D*. *citri* transcriptomes. Most eukaryotes only have one ABCE and three ABCF genes (Table 2). In the phylogenetic tree (Supplementary Fig. S5), DcABCE1 showed the highest homology with BmABC010129 and TcABCE-3A. ABCFs clustered into well-supported separate clades, with DcABCF1 and DcABCF2 located in the HsABCF1 clade, and DcABCF3 and DcABCF4 positioned at the HsABCF2 and HsABCF3 clades separately. Phylogenetic analysis revealed that the ABCE and ABCF subfamilies were highly conserved.

### ABCG subfamily

The ABCG transporter family is present in most metazoan species, fungi and plants. Based on the research of predecessors, ABCG half-transporters were only identified in metazoan species except one ABCG gene in *P*. *xylostella* (Px007949)^51^. However, full-transporters are widely present in fungi and plants^52,53^. The half-transporters have a reverse domain structure with an NBD at the N-terminus and a TMD at the C-terminus (NBD-TMD), while a functional transporter must be dimeric^15^. In humans, five ABCG transporter family genes have been identified. Among these HsABCGs, four HsABCGs except HsABCG2 were mainly involved in the transportation of dietary lipids, while HsABCG2 (breast cancer resistance protein, BCRP) has a series of substrates, including anticancer drugs, and acts as an MRP^54^. Among invertebrates, *D*. *melanogaster* ABCG members were first characterized, including *brown*, *scarlet*, and *white* genes^55^.

**Figure 3:**
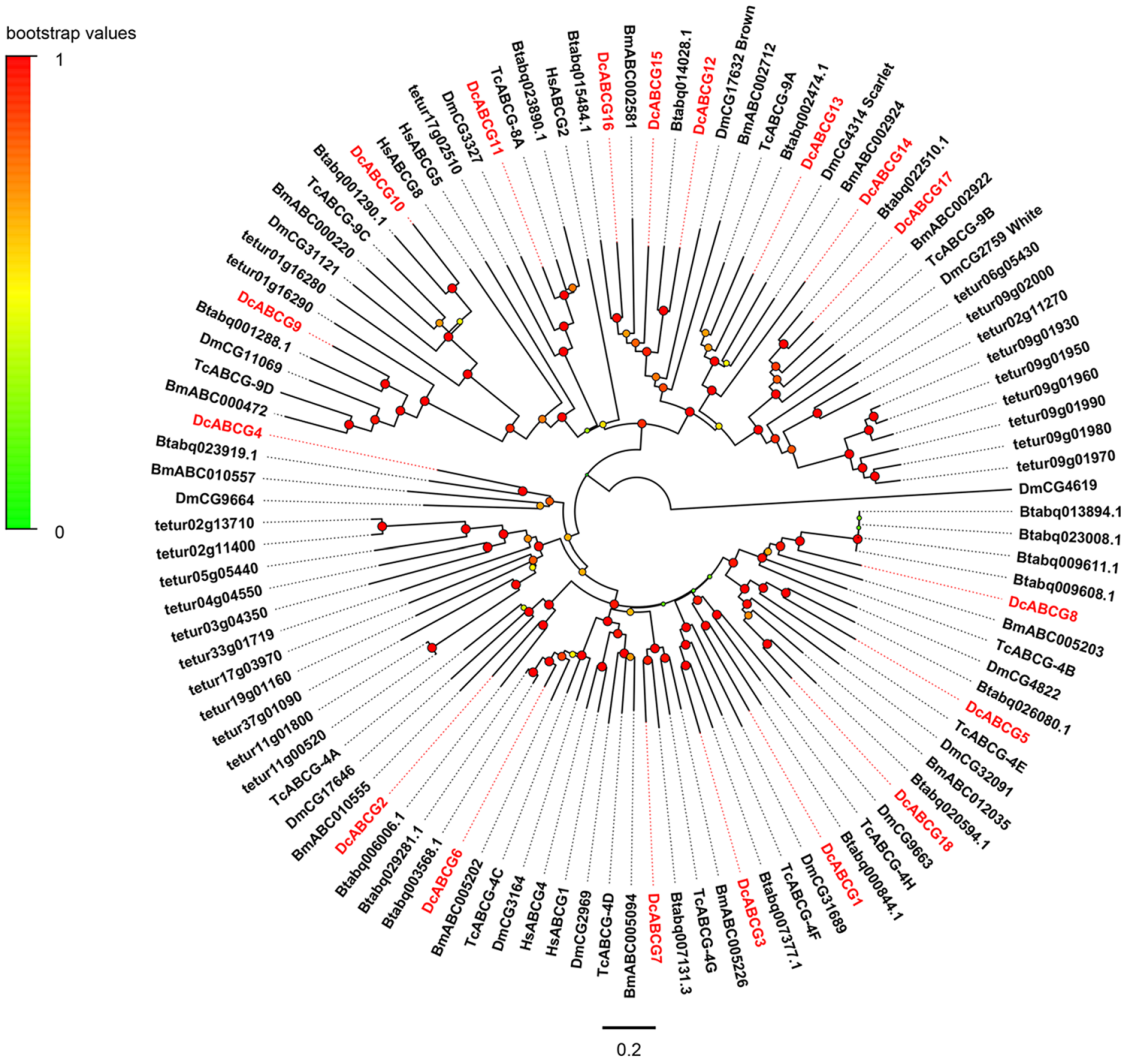
Phylogenetic analysis of ABCG transporters of *D*. *citri* and other species. Dm, *Drosophila melanogaster*; Bm, *Bombyx mori*; Tc, *Tribolium castaneum*; tetur, *Tetranychus urticae*; Btabq, *Bemisia tabaci* Q; Dc, *D*. *citri* (red). The neighbour-joining tree was constructed using MEGA6.0 software and with the Poisson model. The bootstrap values resulted from 1000 replications and are displayed in the size and colour of the circles.

Eighteen ABCG transporter family transcripts were identified in the transcriptomes of *D*. *citri* and represent the largest ABC subfamily in *D*. *citri*, all of which possess full-length coding sequences and are in accord with half-transporters with the topology TMD–NBD. In the phylogenetic tree (Fig. 3), eight *D*. *citri* ABCG genes (DcABCG1-3, DcABCG5-8, and DcABCG18) clustered with potential orthologues of HsABCG1 and HsABCG4 in ABCG clades, where HsABCG1 is involved in regulating the output of cholesterol, while the function of HsABCG4 was not clear^56^. In humans, HsABCG5 and HsABCG8 form a functional heterodimer and play a role in removing plant sterols from the body^56^. In the phylogenetic tree, DcABCG9 and DcABCG10 were two orthologous genes of HsABCG5 and HsABCG8, and all the arthropod orthologues of HsABCG5/HsABCG8 showed a head-to-head arrangement, indicating that DcABCG9 and DcABCG10 may have similar functions as HsABCG5/HsABCG8. Six genes (DcABCG12-17) clustered with *D*. *melanogaster* white, scarlet, and *brown* and the orthologues of the other species. In *D*. *melanogaster*, *white*, *scarlet*, and *brown* are the best-characterized ABCG genes of arthropods, and scarlet or brown takes part in transporting pigment precursors in the Malpighian tubules and relates to the formation of compound eye colour^57,58^. *D*. *melanogaster white* mutants show a white-eye colour phenotype, and this phenomenon has also been confirmed in *T*. *castaneum* and *B*. *mori*^29,59^. However, *white* is also involved in resistance to pesticide, and downregulation of the white orthologues leads to increased Bt resistance in *P*. *xylostella*^60^. In *D*. *citri*, DcABCG17 is orthologous to *white*, DcABCG13 and DcABCG14 are orthologous to *scarlet*, and three genes (DcABCG12, DcABCG15 and DcABCG16) are orthologous to *brown*. DcABCG11 clustered with *B*. *tabaci* Btabq023890.1, *T*. *castaneum* TcABCG-8A, *D*. *melanogaster* DmCG3327, and *T*. *urticae* tetur17g02510. In *D*. *melanogaster*, DmCG3327 (also named E23) is capable of modulating the 20E response^61^, and a similar function has also been found in *T*. *castaneum* TcABCG-8A^29^. DcABCG6 clustered with *T*. *castaneum* TcABCG-4C, *D*. *melanogaster* DmCG3164, *B*. *mori* BmABC005202 and two orthologues of *B*. *tabaci*. In *T*. *castaneum*, TcABCG-4C-dsRNA injected larvae exhibited a rough cuticle as a consequence of desiccation and shrinkage and rapidly caused death during the quiescent stage, in addition, injection of TcABCG-4C-dsRNA into pre-pupae resulted in death at the pupal stage before the pupal-adult molt, while DmCG3164 performs a similar function in *Drosophila*^29,62^.

### ABCH subfamily

**Figure 4:**
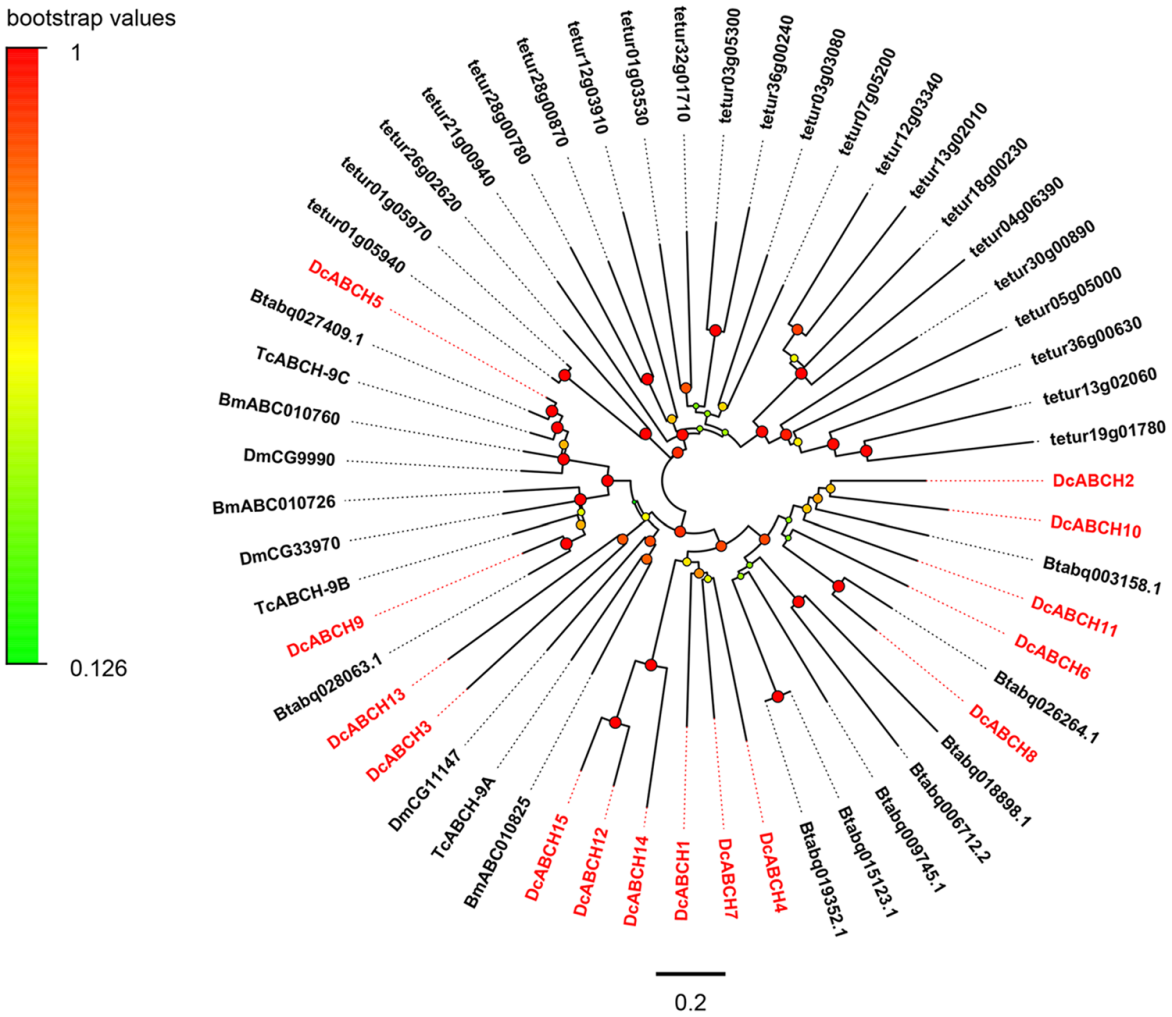
Phylogenetic analysis of ABCH transporters of *D*. *citri* and other species. The abbreviations and colour settings are consistent with Fig. 3. The neighbour-joining tree was constructed using MEGA6.0 software and with the Poisson model. The bootstrap values resulted from 1000 replications and are displayed in the size and colour of the circles.

The ABCH transporter family proteins are half-transporters and share the same NBD-TMD topological structure as the ABCH family. ABCH transporters were first identified in *D*. *melanogaster* and it was then reported that the ABCH subfamily was only found in arthropods, zebrafish *Danio rerio* and marine medaka *Oryzias melastigma*^15,63^. At present, the ABCH subfamily has not been identified in other species such as mammals, plants and fungi^2,52,64^. We identified fifteen DcABCH genes in the transcriptomes of *D*. *citri*, fourteen of them have the full-length ORF. In most insect species, only three ABCH members were found, including *D*. *melanogaster*, *B*. *mori* and *T*. *castaneum* which has an excellent genome sequence, however, a large number of ABCH members were found in three Hemiptera insects (*B*. *tabaci*, *Lygus Hesperus* and *D*. *citri*) and two species of arthropods (*D*. *pulex* and *T*. *urticae*) (Table 2). In the phylogenetic tree (Fig. 4), six DcABCH sequences (DcABCH1, DcABCH4, DcABCH7, DcABCH12 and DcABCH14-15) formed a separate clade, which was similar to the ABCHs of *T*. *urticae*^30^ and *D*. *pulex*^65^, suggesting that the diversity of ABCH proteins in *D*. *citri* has been due to lineage-specific duplications events, this similar expansion was also found in two Hemiptera insects *B*. *tabaci* and *L*. *hesperus*^26,64^.

ABCH plays an important role in insect physiology. In recent years, researchers have been exploring and have a considerable understanding of their physiological function. In *Helicoverpa armigera* and *Manduca sexta*, when the larvae were fed with secondary metabolites, the expression of ABCH subfamily was induced to increase^66,67^. In *D*. *melanogaster*, cold hardening treatment resulted in a 2-fold increase in the expression level of DmCG33970^68^, both DmCG9990 null mutants and RNAi-mediated knockdown DmCG9990 are lethal^62,69^. In addition, DmCG9990 was also found to be associated with the formation of epidermal barrier^70^. In *T*. *castaneum*, injection of dsRNA specific for TcABCH-9C, the ortholog of DmCG9990, resulted in desiccation and 100% larval mortality and a significant reduction in fertility and the egg hatchability. Furthermore, TcABCH-9C dsRNA treated larvae showed a lack of lipids in the epicuticle, and based on these results, the authors inferred that TcABCH-9C was involved in transport of lipids to epicuticle and promoting the formation of waterproof barrier in epicuticle^29^. In a recent study, the ortholog of DmCG9990 in *Locusta migratoria*, LmABCH-9C, were borne out to be associated with transport of lipids to epicuticle and cuticle barrier formation in epicuticle^71^. In *D*. *citri*, DcABCH5 and DcABCH9 are orthologues of DmCG9990 and DmCG33970, respectively (Fig. 4).

### Expression profile of DcABCs

**Figure 5:**
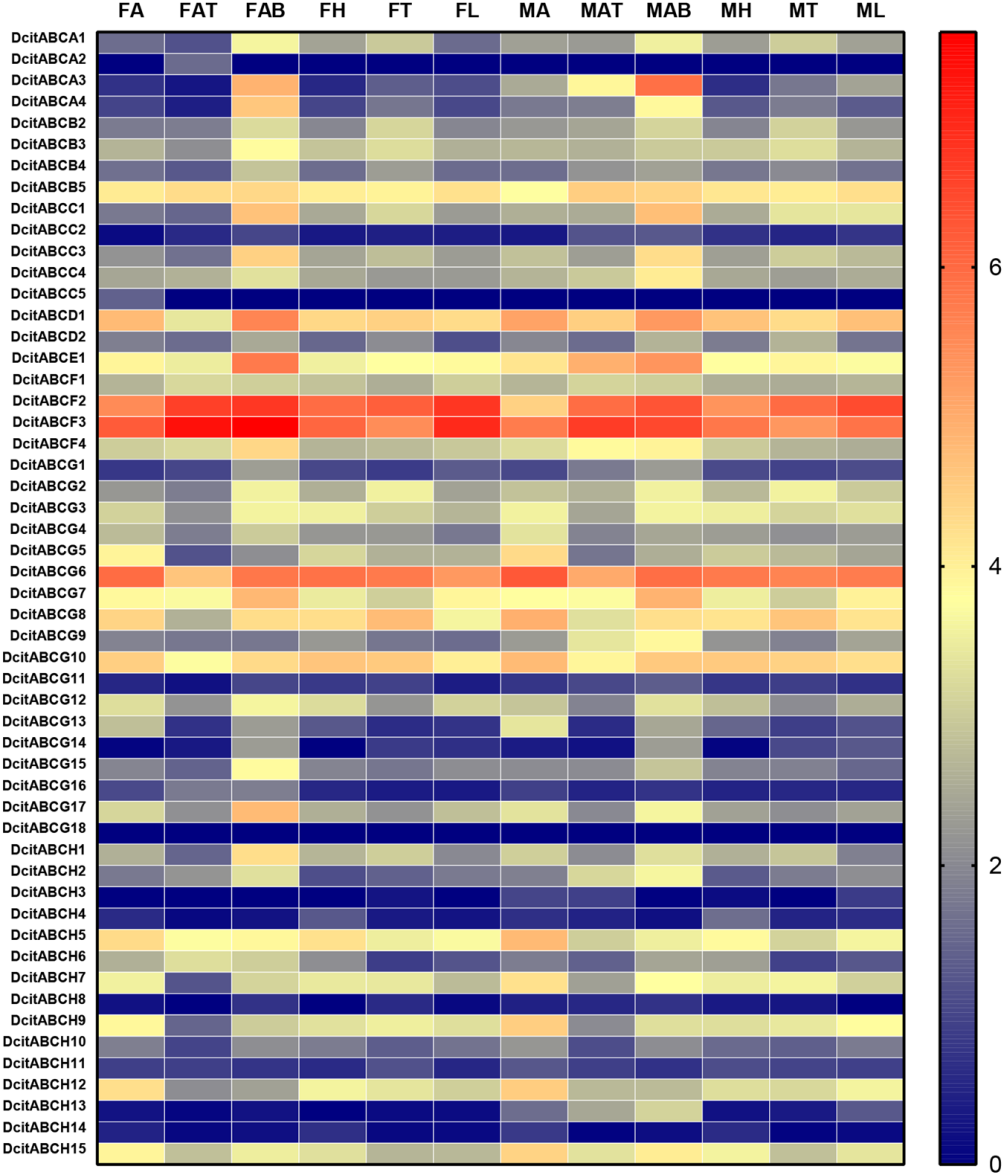
Tissue-specific expression profiles of ABC transporter genes in various tissues of *D*. *citri* based on FPKM values. The mRNA levels, as represented by log2 (FPKM + 1) values, are shown in the heat map with colors ranging from blue (low expression) to red (high expression). MA, male antenna; MH, male head; MT, male thorax; ML, male leg; MAB, male abdomen; MAT, male abdomen terminal; FA, female antenna; FH, female head; FT, female thorax; FL, female leg; FAB, female abdomen; FAT, female abdomen terminal.

**Figure 6:**
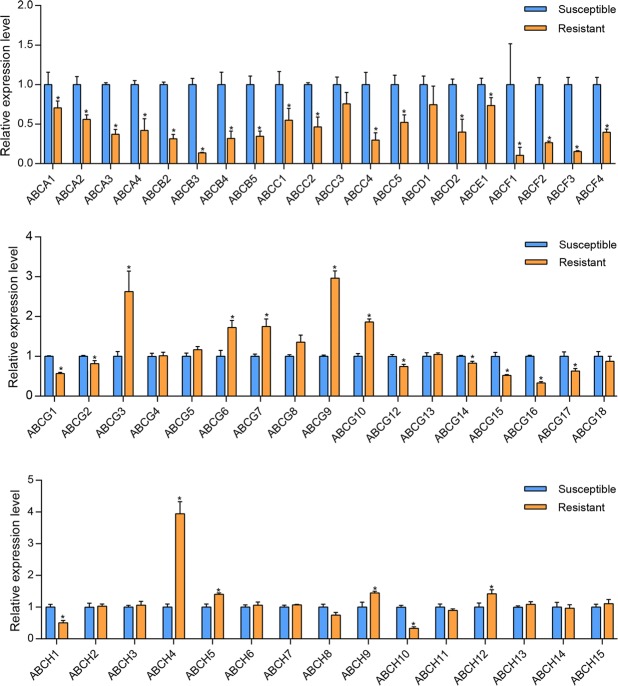
Transcript levels of 52 ATP-binding cassette (ABC) transporter genes in *D*. *citri*. The error bars indicate the standard errors of the means (n = 3), The asterisks indicate significant differences compared with the susceptible strain (Student’s t-test, P < 0.05).

The spatial expression profiles of these ABC transporter genes were estimated by analysing the FPKM values. Ten genes (DcABCB5, DcABCD1, DcABCE1, DcABCF2-3, DcABCG6-8, DcABCG10, and DcABCH5) are widely expressed in adult tissues of *D*. *citri*. Seven ABC transporter genes (DcABCA1, DcABCA3-4, DcABCC1, DcABCC3-4, DcABCG15, and DcABCH2) showed high expression levels in the abdomen of adults (Fig. 5). To understand the possible role of DcABCs in the insecticide resistance of *D*. *citri*, qRT-PCR was used to compare the expression of these genes between the imidacloprid-susceptible and imidacloprid-resistant strains. The expression level of DcABCG11 in susceptible strains was too low to be detected; therefore the expression levels of 52 genes were presented. Nine DcABCs were significantly upregulated in the imidacloprid-resistant strains compared to the susceptible strains (Fig. 6), and the upregulated genes were mainly concentrated in the ABCG and ABCH subfamilies. DcABCH4 was upregulated 3.9-fold in resistant strains, and DcABCG3 and DcABCG9 were upregulated 2.6-fold and 2.9-fold. DcABCG6, DcABCG7, DcABCG10, DcABCH5, DcABCH9, and DcABCH12 were upregulated 1.4-fold to 1.8-fold. Twenty-seven of the 52 DcABCs were down-regulated in resistant strains, and the expression of the remaining 16 DcABCs did not show a significant difference between susceptible and resistant strains.

In arthropods, ABCB, ABCC and ABCG were the three most reported subfamilies associated with insecticide transport or resistance, and the index of insecticide transport or resistance is mainly based on their expression level^15^. In *D*. *citri*, five ABCG transporters were significantly upregulated in imidacloprid-resistant strains, and similar upregulation of expression has also been reported in other insects. For instance, one ABCG transporter was upregulated in the imidacloprid-susceptible *Leptinotarsa decemlineata*^72^. Four ABCG transporters were up-regulated in imidacloprid-treated *B*. *tabaci*^64^. In addition to the ABCG transporter, four ABCH transporters were significantly upregulated in imidacloprid-resistant strains. In the green peach aphid, *Myzus persicae*, an ABCH was upregulated under the stress of pirimicarb^73^, suggesting that ABCH may also be involved in insecticide resistance in insects. The results indicated a potential implication of these genes in imidacloprid resistance. In addition, five of the nine upregulated genes (DcABCG6-8, DcABCG10, and DcABCH5) were expressed widely in adult tissues, which may demonstrate that the wide expression of ABC transporters may contribute to the transport of exogenous substances such as pesticides. Twenty-seven DcABCs were downregulated in resistant strains. Similarly in *P*. *xylostella*, Px006766 (PxABCF3) and Px013659 (PxABCA12) were downregulated in chlorpyrifos-resistant and fipronil-resistant strains^51^. This may indicate that not all ABCs are involved in detoxification and may be a physiological adaptation to long-term pesticide pressure.

## Conclusions

The major objective of this study was to identify the ATP-binding cassette transporter gene family in *D*. *citri*. In this study, fifty-three genes encoding ABC transporters were identified in *D*. *citri* using RNA-Seq and transcriptomic analysis. Among 8 subfamilies, ABCG and ABCH have more members in *D*. *citri*. Moreover, nine genes of these two subfamilies were upregulated in the imidacloprid-resistant strain of adult *D*. *citri* and five of them were expressed extensively in adult tissues. These results enrich the research content regarding the insecticide resistance mechanism in *D*. *citri* and will further facilitate our understanding of imidacloprid-resistance mechanisms in this pest.

## Materials and Methods

### Insect rearing and strains

Two strains of *D*. *citri* were used in this study: laboratory-susceptible strains and imidacloprid-resistant strains. The laboratory susceptible-strains were collected from *Murraya exotica* on the campus of South China Agricultural University, Guangzhou, Guangdong Province, China, in 2013, and this population was reared in the greenhouse without exposure to any insecticides. The imidacloprid-resistant strains originate from field populations in Guangzhou, Guangdong Province, China, in 2016, and then they were continuously exposed to imidacloprid to select the resistant strains. A 52.19-fold imidacloprid-resistant strain was obtained through 9 generations of continuous selection via the leaf dip method^25^. Both strains were kept rearing on M. exotica in a climate chamber (26 °C, 80% RH) and a 14:10 h (light:dark) photoperiod.

### Sample collection and RNA Seq

The tissues of insects were dissected from 3-day-old adults of laboratory-susceptible strains. A total of 2000 antennas (including a modicum of tissues of heads), 200 heads (antennas removed), 150 thoraxes, 300 legs, 150 abdomens, and 1000 terminal abdomens (cut from the 5th abdominal segments) were collected from male adults, and the tissues from famale adults had equal numbers. Total RNA from each sample was extracted using TRIzol Reagent (Invitrogen, Waltham, MA, USA). Total RNA samples were quantified and assessed for quality by a NanoDrop 2000 (Thermo Fisher Scientific, Waltham, MA, USA). Transcriptome sequencing was performed on an Illumina HiSeq. 2500 platform (Genewiz, Suzhou, China), and a total of 143.37 Gb of raw data was acquired. After removing low-quality, adaptor and contaminating sequence reads, 137.22 Gb of clean reads was obtained. The clean data were assembled by Trinity, and 297,614 unigenes larger than 200 bp were obtained, the unigenes were submitted to InsectBase (http://www.insect-genome.com/data/Diaphorina_citri.transcript.fa.tar.gz)^74–76^. The raw data of the transcriptomes were submitted to the NCBI Short Read Archive (SRA) database (Submission ID: SRP139008) (https://www.ncbi.nlm.nih.gov/sra/SRP139008)^76^.

### Identification of ABC transporters in *D*. *citri*

The local blast program of BioEdit software was applied to identify candidate ABC transporter genes. The amino acid sequences of *H*. *sapiens*^2^, *D*. *melanogaster*^30^, *B*. *mori*^47^, *T*. *castaneum*^29^, *B*. *tabaci*^64^, *P*. *xylostella*^51^, *T*. *urticae*^30^, and *Saccharomyces cerevisiae*^77^ were used as BLAST queries with an E-value threshold of 10^−5^. To obtain the whole ABC transporter genes as far as possible, the same method was also used to identify ABC transporters in the genome of *D*. *citri* (Accession: GCF_000475195.1). The candidate ABC transporter genes were reconfirmed by BLASTx analysis with the non-redundant protein sequence (NR) of NCBI (http://www.ncbi.nlm.nih.gov/).

### Protein structure and domain prediction

The open reading frames (ORFs) of the candidate ABC transporter genes were predicted using the ORF finder (http://www.ncbi.nlm.nih.gov/). The ORFs of the genes in each subfamily were aligned using ClastalW to search alternative splicings to confirm the isoforms from the same gene. The NBD and TMD of each potential ABC transporter genes were verified by searching with the Pfam program (http://pfam.xfam.org/)^78^ and Conserved Domains (http://www.ncbi.nlm.nih.gov/)^79^. The domains were graphed with the illustrator program DOG 2.0.1^80^. N-glycosylation sites and O-glycosylation sites were predicted using NetNGlyc 1.0 (Potential Score > 0.5, Jury agreement: 9/9) (http://www.cbs.dtu.dk/services/NetNGlyc/) and O-glycosylation^81^ (G-score > 0.5) (http://www.cbs.dtu.dk/services/NetOGlyc-3.1/) respectively.

### Phylogenetic analysis

To classify the position of *D*. *citri* ABCs within ABC classes (A-G), the amino acid sequences of NBDs of *D*. *citri* ABC transporters were used to resolve their phylogenetic relationships. When a protein had two NBDs, the N-terminal NBD was used. To analyse the evolutionary placement of ABC transporters in *D*. *citri*, comparison analyses among each subfamily of ABC transporters from *D*. *citri*, *D*. *melanogaster*, *B*. *mori*, *T*. *castaneum*, *B*. *tabaci*, and *T*. *urticae* were conducted, and the full-length protein sequences were subjected to phylogenetic analyses (Supplementary data). Sequences were aligned by the ClustalW alignment algorithm^82^, and MEGA6 was used to construct the neighbor-joining trees with the Poisson model and 1,000 bootstrap replicates^83^, the dendrograms were viewed in FigTree and edited in Adobe PhotoShop CS6.

### Expression analysis of ABC transporters

Gene expression levels for each tissue sample were estimated by RSEM (RNA-Seq by Expectation-Maximization) (v1.2.6)^84^. The spatial expression of these ABC transporter genes was estimated using the Fragments Per Kilobase of transcript per Million fragments (FPKM) method, which is based on the number of uniquely mapped reads^85^. The FPKM values of each gene were transformed into log2 (RPKM + 1) values, and GraphPad Prism 7.01 was used to generate and visualize the expression profile.

The relative expression of ABC transporter mRNAs in imidacloprid-susceptible and imidacloprid-resistant strains was determined using quantitative real-time PCR (qRT-PCR) with SYBR-green fluorescence. Total RNA was extracted from two strains using TRIzol Reagent (Invitrogen, Waltham, MA, USA). The PrimeScript™ RT reagent Kit with gDNA Eraser (Takara, Dalian, China) was used for cDNA synthesis, and two reference genes, namely, β-actin (XM_008473151) and α-tubulin (DQ675550), were used as internal controls^86,87^. The gene-specific primers were designed by the Primer 3 program (http://elixir.ut.ee/Main/Services) (Supplementary Table S1). Gene-specific primers synthesis were completed by TsingKe Biotech Co., Ltd (TsingKe, Beijing, China). The CFX96 Real-Time PCR System (Bio-Rad, Hercules, CA, USA) and the Go Taq® qPCR Master Mix (Promega, Madison, WI, USA) were used to perform qRT-PCR reactions. Finally, the relative values of mRNA expression were calculated by The 2^−ΔΔCt^ method^88^, and the expression level of imidacloprid-susceptible strain was used as the calibrator. The significance of differences between two strains was determined using Student’s t test (P < 0.05). Three biological replicates were analysed for each experiment. A total of 120 ACP adults (three biological replicates, n = 40) from susceptible and resistant strains were used for qRT-PCR analysis, and three technical replicates were performed in each qRT-PCR reaction.

## Supplementary information

Supplementary information

Dataset 1
