# Supplementary material for: Identification of candidate ATP-binding cassette transporter gene family members in Diaphorina citri (Hemiptera: Psyllidae) via adult tissues transcriptome analysis
Source: Sci Rep. 2019 Nov 1;9:15842. doi: 10.1038/s41598-019-52402-3 (PMC6825165; doi:10.1038/s41598-019-52402-3)
Supplement: Supplementary file 1 — Supplementary information [file 41598_2019_52402_MOESM1_ESM.docx]

**Supplementary Information:**

**Identification of candidate ATP-binding cassette transporter gene family members in *Diaphorina citri* (Hemiptera: Psyllidae) via adult tissues transcriptome analysis**

Zhengbing Wang^1^, Fajun Tian^1^, Lijun Cai^2^, Jie Zhang^2^, Jiali Liu^1^ and Xinnian Zeng^1,^*

^1^Guangdong Engineering Research Center for Insect Behavior Regulation, Key Laboratory of Bio-Pesticide Innovation and Application, College of Agriculture, South China Agricultural University, Guangzhou 510642, China

^2^State Key Laboratory of Ecological Pest Control for Fujian and Taiwan Crops, Institute of Applied Ecology, Fujian Agriculture and Forestry University, Fuzhou 350002, China

^*^Corresponding author: Professor Xinnian Zeng, Fax: +86-20-85288356. E-mail: zengxn@scau.edu.cn

This file includes:

Table S1-S2

Figures S1-S5

| Gene | Forward Primer sequence (5’ to 3’) | Reverse Primer sequence (5’ to 3’) |
| --- | --- | --- |
| DcABCA1 | ACCCAGCTCTGATCCTGAAC | GCTTGGCTCGATTCTCACTG |
| DcABCA2 | CAATTCTCTCGTCACGGCATT | CCAGAGTACCATGCCCACTAT |
| DcABCA3 | GGCGTGGTCTGTGGGATATA | CTGTAGCCTTGCCCGAATTC |
| DcABCA4 | ATTCCCTACGTCCAGTTGCA | ATCCCTTCCTTCAGCTTGCT |
| DcABCB2 | CTAAACCGAGTGTGTGCCAG | GGTTCGGATTGCTTGGTGTT |
| DcABCB3 | TGTTGGAATCTACACGGCCT | GCGCTCTGACCAAAGTTCAA |
| DcABCB4 | ACTGGTCGTGTCTCAAGGTT | CACGTGATAGAGAGCGGAGT |
| DcABCB5 | TCCAAGTAGACCAGATGCTGT | GCTCCTTTATGTCAACGCCA |
| DcABCC1 | ACACGGGGCTTCACAATAGA | GTCCTTGTTCTCCAGAGGCT |
| DcABCC2 | ACAAGATGTGGCAGGAGGAA | TGCCCAAAATATGCCATCCG |
| DcABCC3 | TGGTCACGGCCTACTTCAAT | GGGAAGAGGGAGCTTTGTCT |
| DcABCC4 | GCAAGTCATGTGTTCAGCCA | GCGCTAGGTCGTTGTAGTTG |
| DcABCC5 | CACTACCGCCACAGAGACAG | CCAGCTTGAGACACTTCCGA |
| DcABCD1 | AAGGAGATCGTCGAGCTAGC | ACTCCCGTCTAGACTGTCCT |
| DcABCD2 | GTGTTGGCAGGGAGAGAGAT | CTTGGCTTTGAACCTTCGCT |
| DcABCE1 | AGGACAGTTCACGGACTCTG | GAGTTGTCGCACCAAACCTT |
| DcABCF1 | GCAAACCTACAGATCGCCAG | TCCCGATTCATCACGTTCCA |
| DcABCF2 | ACTGCTGAAGAAACACCTGC | TACCCTTGCCTTCTGTCCTC |
| DcABCF3 | CCGGAAAGAGTACCCTGCTA | CAGTCAGACCATACCTGCCA |
| DcABCF4 | ACACTTCTCTGGAGGATGGC | TGGAAGATGTCGGTGGGTAC |
| DcABCG1 | AATTGTCCGTCATCCTGGGT | AGATTCTTCCACCGTCAGCA |
| DcABCG2 | CGACTCTCTCTATGCCCTGG | TAACCGTATCCGCCCCATAC |
| DcABCG3 | GTCACAGGGGAATACGGACA | TGAGTTACCGTCCAGTCTCG |
| DcABCG4 | CGCTGTTGAACATTTTGACCG | AGTGAGGGGTCCAACTTTAGC |
| DcABCG5 | GTCCCTACTTGTAGCAGCACA | AGGAGGGTTGTTGACGAGTTC |
| DcABCG6 | ATCTGTACACCTTGGCCGAA | CTGACTTGGGGAACGGTTTG |
| DcABCG7 | GGGTCAGAAGAAGAGGCTGT | TTGTCCGCCAGATAGCAGAT |
| DcABCG8 | TGGACAAGATGTAGTGCCGT | TTGTTGGGCGAGAGGTAGTT |
| DcABCG9 | TGGTCATGCTTTCTGTTCGC | ACAAGCCACTCTGCCATACT |
| DcABCG10 | AACCACTGAATCCACGCATG | CTCAAGACCTCTGACCTGCA |
| DcABCG12 | AGTGATGGAGAACTTGGGCA | CACACGCCAGTTGTTTGAGA |
| DcABCG13 | CCCTGATCAACTCACTGCTGA | GAAGTAAAGCTGGGTCCGTCA |
| DcABCG14 | ATGATGGGCTCGCTCTACTC | CTCAGGAACAGCGGGAATTG |
| DcABCG15 | CGTCGCTGTATTGTGGCTAC | GAAACTGTCCAGCCCTGTTG |
| DcABCG16 | TTCACGAACACATGACGCTC | AGATGGGTCGGTCAACAACT |
| DcABCG17 | CACTCTCCTCAACACCCTCAC | CCTCCACTCTCTGCATTCGTT |
| DcABCG18 | CTACCTAGCGACACACCCAAG | GTTGATGAGTTCGACGGCAAT |
| DcABCH1 | TCAGCAAGAAACAGTTGGGC | GCCACTGAGAGATCCACACT |
| DcABCH2 | CAAGAACATTGCCTGGGGTC | CGCCTCCACCAGACTTTCTA |
| DcABCH3 | CGACCTCTGAACACGGGATT | CGTTCGGTAGTGTGGTTCCA |
| DcABCH4 | CTTGGTAGCAGGTCTCACGA | AGTGCCACAAATGCCTTCAG |
| DcABCH5 | GAGATCCGAGTCTGGCTTGA | GCGACGAAATCAGTGAAGCT |
| DcABCH6 | AGCTATGATACCCCGAACGG | CCAGATGTTGTTGTGCCACA |
| DcABCH7 | TGGAAGAGTCAGAGCCACAG | ATTCATTGCTCACCAGGGGA |
| DcABCH8 | TCATGTGCTTCCTCCTCCTG | TCTCCACGTATCGACAGCTC |
| DcABCH9 | GGAAGACAACCCTCCTCTCC | ACCGTTGATCCATCCGAAGT |
| DcABCH10 | TAAGACCACCCTGTTGCACT | TCGCTGGGCAGTTCTAAGAA |
| DcABCH11 | TGTCAAGGATGGGCAAAACG | TCTGTTTGGAGGCGTTGTTG |
| DcABCH12 | CCATGCTGGCCCACTATTTC | CTGTCAACACCGTAACTGGC |
| DcABCH13 | TGCCACAAGAACTAGCCATGT | TGGGGCTTATGAAGAATCGCT |
| DcABCH14 | TGATGAGACAAGGCGTGCTT | TGAGGCCACACAGCACTTAG |
| DcABCH15 | CGGTTTCATGCCTCAGGAGA | ACTTGACCGCCACTTAACGT |
| β-Actin | TGTGACGAAGAAGTTGCTGC | TGGGGTATTTCAGGGTCAGG |
| α-Tubulin | CTCTACAGGGGTGACGTTGT | TTGGAAAGCATACACACGGC |

**Table S1.** Sequences for quantitative real-time PCR (qRT-PCR) primers.

| **Gene** | **ABC transporters in the genome of *D. citri***  **(Accession no.)** | **Gene** | **ABC transporters in the genome of *D. citri***  **(Accession no.)** |
| --- | --- | --- | --- |
| DcABCA1 | XM_008487540.2  XM_008489408.2  XM_008489414.1  XM_008489415.1  XM_008489418.1  XM_017449593.1 | DcABCG8 | XM_017442304.1 |
| DcABCA2 | XM_017442682.1  XM_017442683.1 | DcABCG9 | XM_008471638.2 |
| DcABCA3 | XM_008482446.2  XM_008485750.1 | DcABCG10 | XM_008471628.1  XM_008489027.1  XM_017449401.1 |
| DcABCA4 | XM_017444516.1 | DcABCG11 | XM_008470778.2  XM_008470789.2  XM_017448539.1 |
| DcABCB2 | XM_008475819.1  XM_017444859.1  XM_017448417.1 | DcABCG12 | XM_008488702.2  XM_008488730.2 |
| DcABCB3 | XM_008471014.2  XM_008485420.1 | DcABCG13 | XM_017443579.1  XM_017443582.1 |
| DcABCB4 | XM_017448497.1 | DcABCG14 | XM_017444431.1  XM_017444433.1 |
| DcABCB5 | XM_008475795.2 | DcABCG15 | XM_017445317.1 |
| DcABCC1 | XM_008474062.2  XM_017442517.1 | DcABCG16 | XM_017447000.1 |
| DcABCC2 | XM_008476464.1  XM_008486812.2  XM_017445095.1 | DcABCG17 | XM_008469724.2 |
| DcABCC3 | XM_008485877.1  XM_017444645.1 | DcABCG18 | XM_008478449.1 |
| DcABCC4 | XM_017448532.1 | DcABCH1 | XM_008479000.1 |
| DcABCC5 | XM_017443225.1 | DcABCH2 | XM_017449873.1 |
| DcABCD1 | XM_008475201.1  XM_008475202.1 | DcABCH3 | XM_008489117.2  XM_008489166.1  XM_008489167.1 |
| DcABCD2 | XM_008485242.2  XM_017443396.1 | DcABCH4 | XM_017442443.1 |
| DcABCE1 | XM_008486216.2  XM_008486217.2  XM_017446038.1  XM_017448883.1 | DcABCH5 | XM_008484624.1  XM_008487033.2 |
| DcABCF1 | XM_008485741.2  XM_017449902.1 | DcABCH6 | XM_017448455.1 |
| DcABCF2 | XM_008471797.2 | DcABCH7 | XM_017446333.1 |
| DcABCF3 | XM_008469569.1 | DcABCH8 | XM_017446950.1 |
| DcABCF4 | XM_008471988.2  XM_008481781.2 | DcABCH9 | XM_017444163.1  XM_017444164.1 |
| DcABCG1 | XM_017445834.1  XM_017445837.1 | DcABCH10 | XM_017449874.1  XM_017449877.1 |
| DcABCG2 | XM_017444962.1 | DcABCH11 | XM_017445680.1 |
| DcABCG3 | XM_008481115.1 | DcABCH12 | XM_017444627.1  XM_017448359.1 |
| DcABCG4 | XM_008471938.2  XM_017443339.1 | DcABCH13 | XM_008475190.1  XM_008486720.2 |
| DcABCG5 | XM_017446283.1 | DcABCH14 | XM_017443111.1 |
| DcABCG6 | XM_008484627.2  XM_008485553.1 | DcABCH15 | XM_017445606.1 |
| DcABCG7 | XM_017444252.1 |  |  |

**Table S2.** The match information of ABC transporter genes from transcriptome and genome.


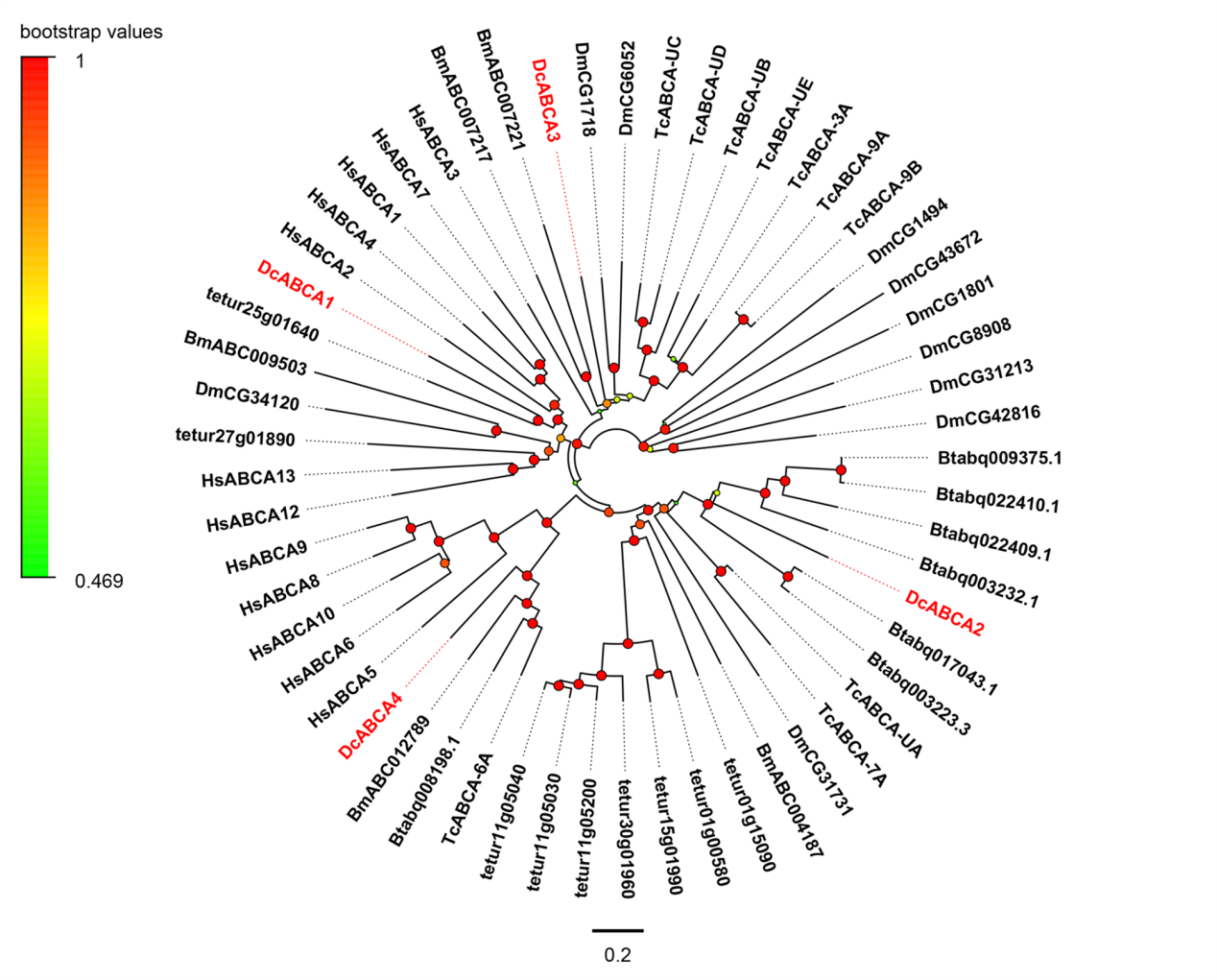


**Figure S1.** Phylogenetic analysis of ABCA transporters of *D. citri* and other species. Dm, *Drosophila melanogaster*; Bm, *Bombyx mori*; Tc, *Tribolium castaneum*; tetur, *Tetranychus urticae*; Btabq, *Bemisia tabaci* Q; Dc, *D. citri* (red). The neighbour-joining tree was constructed using MEGA6.0 software and with the Poisson model. The bootstrap values resulted from 1000 replications and are displayed in the size and colour of the circles.


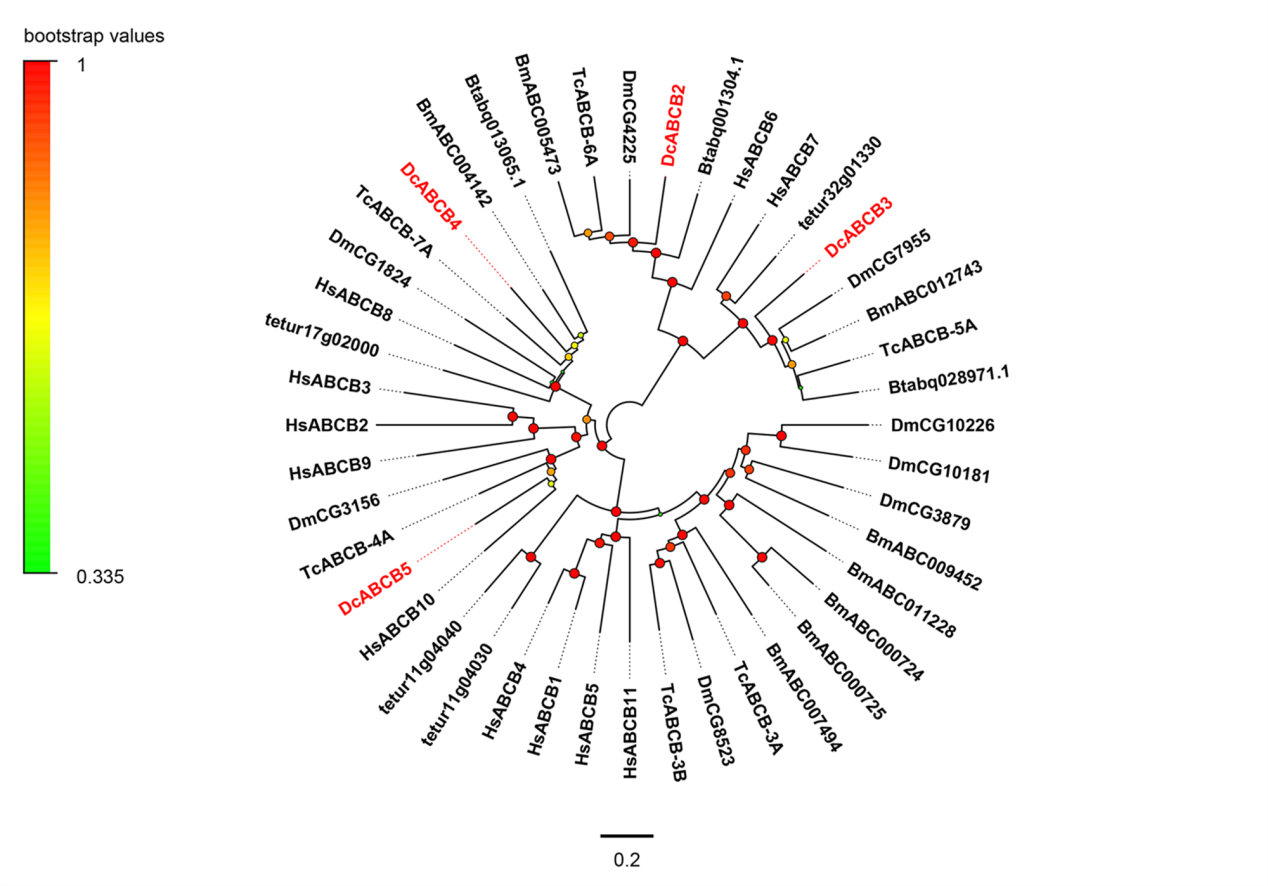


**Figure S2.** Phylogenetic analysis of ABCB transporters of *D. citri* and other species. The abbreviations and colour settings are consistent with Figure S1. The neighbour-joining tree was constructed using MEGA6.0 software and with the Poisson model. The bootstrap values resulted from 1000 replications and are displayed in the size and colour of the circles.


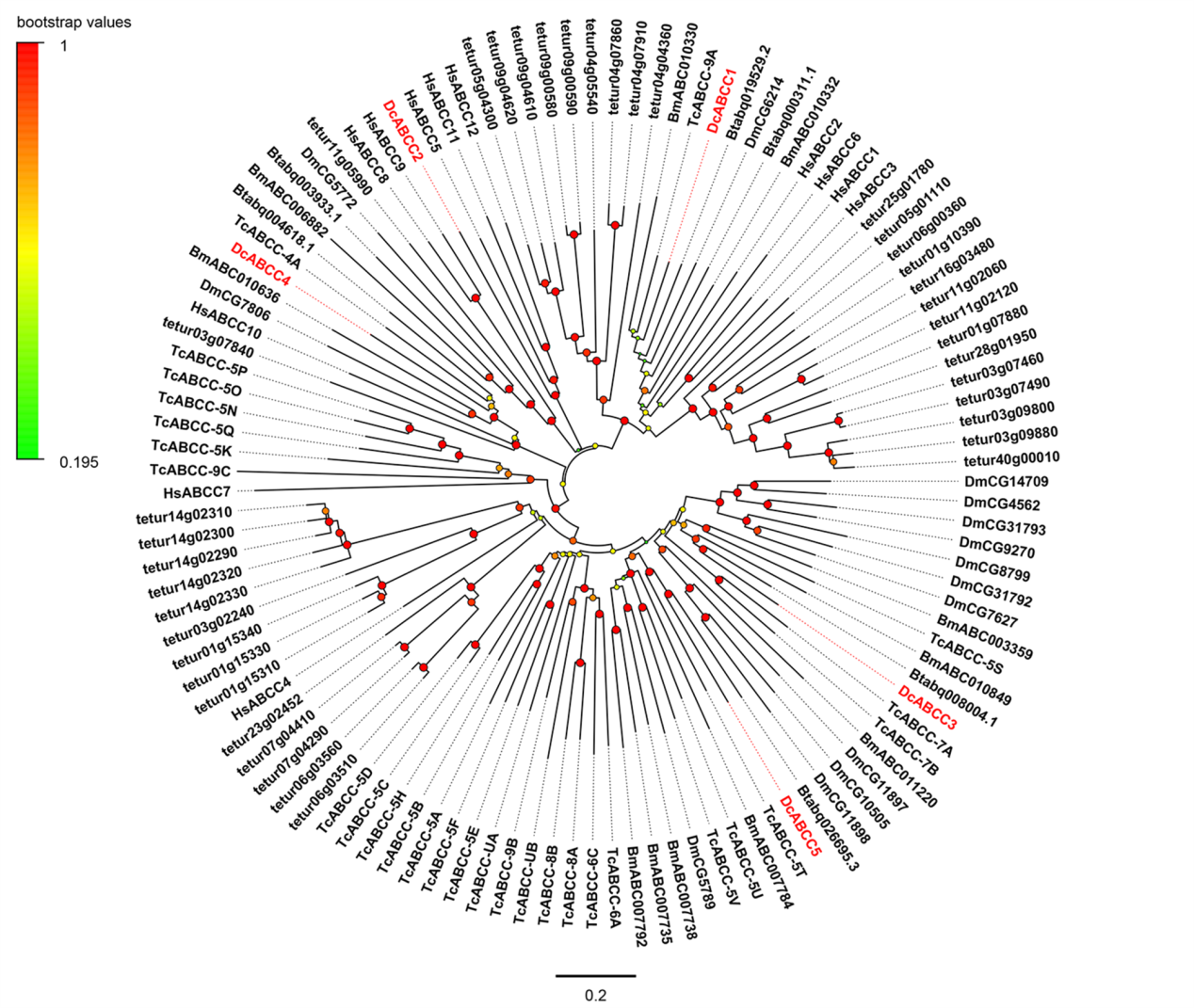


**Figure S3.** Phylogenetic analysis of ABCC transporters of *D. citri* and other species. The abbreviations and colour settings are consistent with Figure S1. The neighbour-joining tree was constructed using MEGA6.0 software and with the Poisson model. The bootstrap values resulted from 1000 replications and are displayed in the size and colour of the circles.


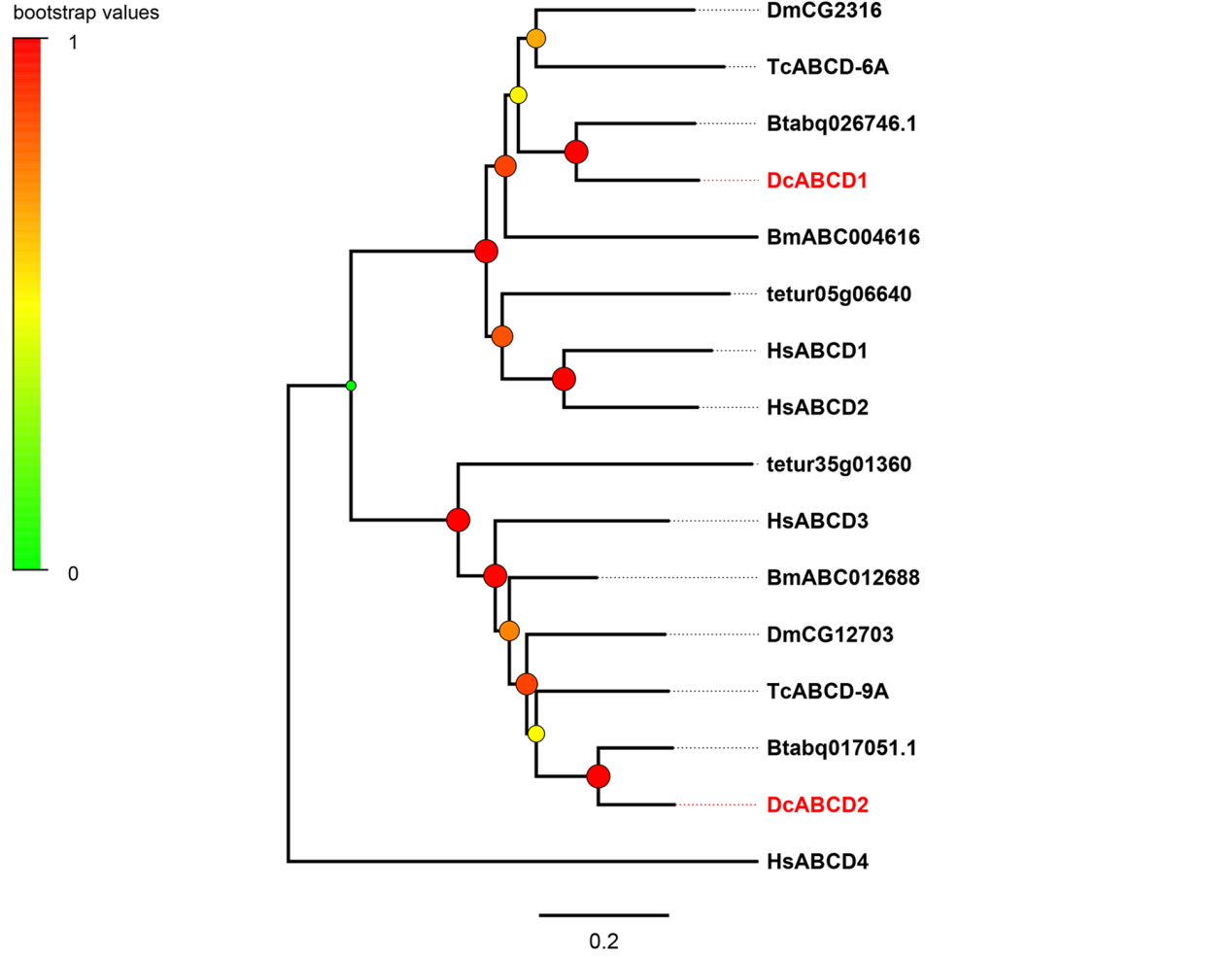


**Figure S4.** Phylogenetic analysis of ABCD transporters of *D. citri* and other species. The abbreviations and colour settings are consistent with Figure S1. The neighbour-joining tree was constructed using MEGA6.0 software and with the Poisson model. The bootstrap values resulted from 1000 replications and are displayed in the size and colour of the circles.


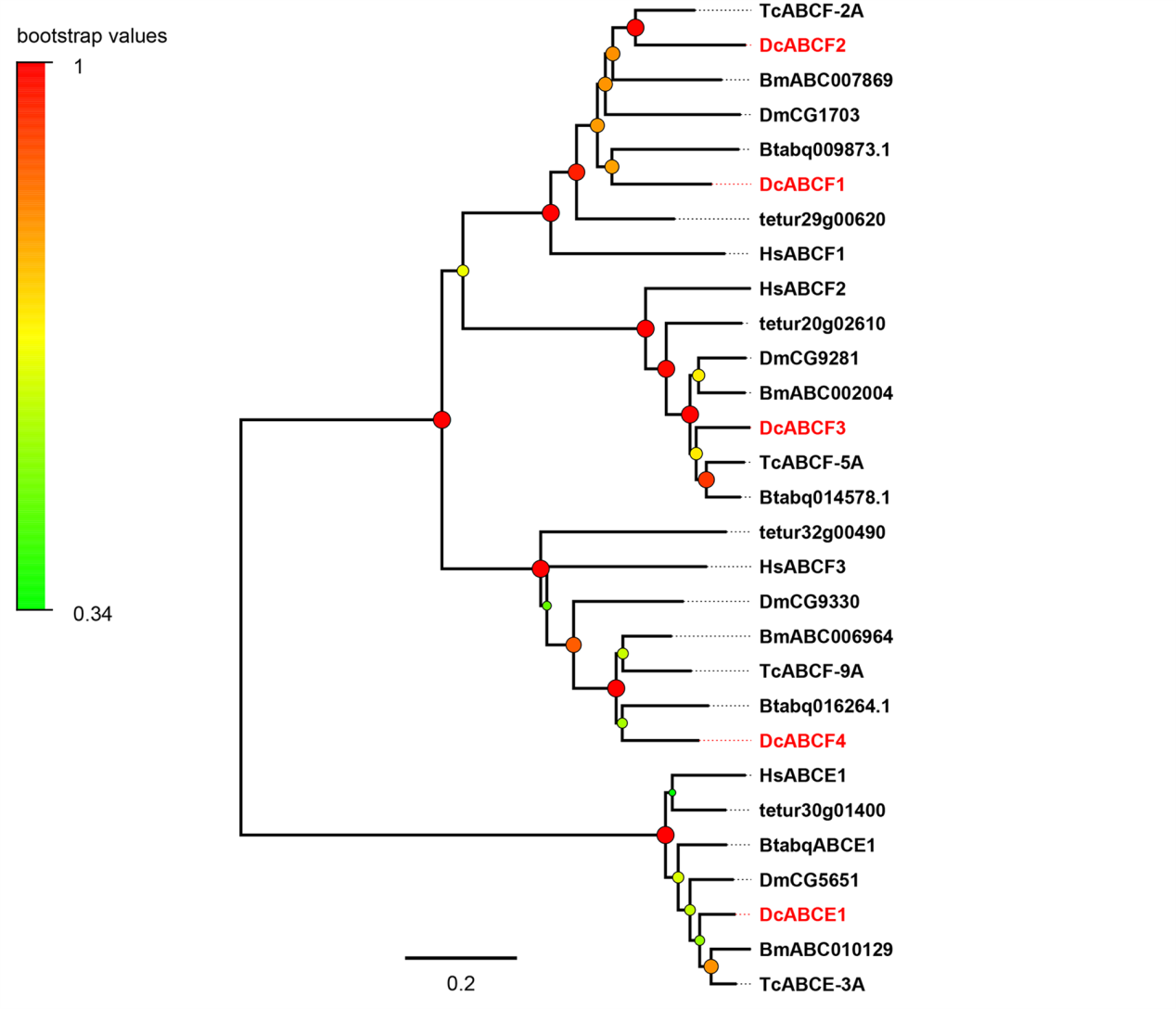


**Figure S5.** Phylogenetic analysis of ABCE and ABCF transporters of *D. citri* and other species. The abbreviations and colour settings are consistent with Figure S1. The neighbour-joining tree was constructed using MEGA6.0 software and with the Poisson model. The bootstrap values resulted from 1000 replications and are displayed in the size and colour of the circles.
